# Active methodologies association with online learning fatigue among medical students

**DOI:** 10.1186/s12909-022-03143-x

**Published:** 2022-02-01

**Authors:** Juliana Barros de Oliveira Kubrusly Sobral, Danilo Lopes Ferreira Lima, Hermano Alexandre Lima Rocha, Elias Silveira de Brito, Lara Hannyella Goveia Duarte, Loren Beatriz Bastos Braga Bento, Marcos Kubrusly

**Affiliations:** 1 Unichristus University Center, Fortaleza, CE Brazil; 2 Department of Global Health and Population, Harvard T. H. Chan School of Public Health, Boston, MA USA

**Keywords:** Education, Medical, Fatigue, COVID-19, Education, Medical, Undergraduate

## Abstract

**Background:**

Due to the current scenario of the COVID-19 pandemic and the social distancing issues, distance learning was implemented in many medical schools. Educational institutions faced the challenge of continuing to promote teaching and learning while keeping teachers and students in their homes, aiming to reduce the spread of the virus. This change compromised the students’ mental health, due to the degree of exhaustion or fatigue attributed to the involvement in videoconferences, called “zoom fatigue”. Despite the importance of zoom fatigue for medical education, it can be observed that there have not been studies on the role of the online teaching and learning process through active methodologies in the genesis of this fatigue. We aimed to assess the association of the teaching method used and the prevalence of zoom fatigue.

**Methods:**

A cross-sectional, quantitative, analytical study was carried out in Medical Schools of Ceará, Brazil. Problem-Based Learning (PBL) teaching methodology is the only methodology used in the first semester and PBL together with traditional teaching, i.e., hybrid teaching, is used in the other ones. The Zoom Exhaustion & Fatigue Scale (ZEF) was used, with the questions currently validated for Brazilian Portuguese. Chi-square tests were used to verify the statistical association between the measured variables and the teaching methodology.

**Results:**

The prevalence of zoom fatigue reached 56% in students using the hybrid model, versus 41% in those using the PBL methodology, with a statistically significant difference (*p* value = 0.027). The mean prevalence of overall zoom fatigue was 48%. Students using the hybrid methodology differed from PBL students by having a significantly higher frequency of feelings of wanting to be alone after a videoconference (16.9 vs. 7.1%, respectively) and needing time to be alone after a video conference (10.2 vs. 3.6%, respectively).

**Conclusions:**

Considering that zoom fatigue may stay with us for years beyond the COVID-19 pandemic, it is important to know and provide instructions on how to reduce video conferencing fatigue. The present study suggests that the active participation of students and the number of activities are important factors to be considered.

## Introduction

Due to the current scenario of the COVID-19 pandemic and the social distancing issues, Distance Learning in its Remote Learning modality started to have a new meaning in the context of national education, consolidating itself as a new territory to be explored. In this scenario, educational institutions faced the challenge of continuing to promote teaching and learning while keeping teachers and students in their homes, aiming to reduce the spread of the virus. In Brazil, as in many other countries, in-person classes were suspended and, without prior planning, all schools in the world temporarily switched to an alternative mode of online teaching called ‘Emergency Remote Teaching’ [1–3]. Therefore, all activities started to be developed through videoconferences, mobile applications and computer programs. Tools such as Zoom, Google Hangouts, Skype, among others, have become crucial for the continuity of education.

This change compromised the teaching and learning process and the students’ mental health, due to the degree of exhaustion or fatigue attributed to the involvement in videoconferences, called “zoom fatigue” [4, 5]. Considering the possible eventualities that lead to this fatigue caused by the exaggerated videoconferencing use, studies have shown that being in a videoconference requires more focus than if one is attending the event in person. Video chat use means we need to work harder to process nonverbal cues, such as facial expressions, voice pitch and tone, as well as body language. Paying more attention to online events consumes a lot of energy, which causes people to have conflicting and exhausting feelings [6].

It should be noted that it is not simply the exaggerated videoconferencing use that can lead to zoom fatigue. In this aspect, Kaplan, in his Attention Restoration Theory (ART) explains how human energy is consumed and postulates that, in meetings where individuals have the perception of belonging to the group (“compatibility”), they feel more connected with each other and motivated (“soft fascination”), they are associated with a lower degree of fatigue [7, 8]. Therefore, wouldn't students participating in videoconferences experience less zoom fatigue during student-centered curricular models such as “Problem based learning” (PBL) than those who use the curriculum based on hybrid learning (PBL and traditional teaching)?

Despite the importance of zoom fatigue for medical education, it can be observed that there have not been studies on the role of the online teaching and learning process through active methodologies in the genesis of this fatigue. Hence, to fill up this gap, a cross-sectional study of medical students from different institutions and of different teaching models was developed to assess the association of the teaching method used and the prevalence of zoom fatigue.

## Methods

### Study design

A cross-sectional, quantitative, analytical study was carried out in Medical Schools of Ceará, Brazil. Problem-Based Learning (PBL) teaching methodology is the only methodology used in the first semester of one of the evaluated universities and PBL together with traditional teaching, i.e., hybrid teaching, is used in the other ones. Ceará is an impoverished state of Brazil, with a monthly *per capita* income of around US$ 172.00 dollars, a frequent scenario in developing countries, although all included students have access to classes via the internet. The study period went from May 2021 to June 2021.

### Study population and sample

All enrolled students aged over 18 years of age, of both genders, who are linked to higher education institutions and attending courses in the health area were included in the study. Students who did not use virtual platforms during the pandemic were excluded.

### Data collection

Data were collected using electronic Google forms, sent directly to the students.

### Variables

The English version of the Zoom Exhaustion & Fatigue Scale (ZEF) was used [9], with the questions currently validated for Brazilian Portuguese [10]. This scale consists of a set of fifteen questions that are divided into five domains: overall, visual, social, motivational and emotional, and aims to understand some of the physiological effects and mechanisms involved in large-scale participation in videoconferencing. In the original article, ‘Zoom Fatigue’ was defined as the fatigue that can be experienced during or after participating in a videoconference. The teaching methodology which the student was experiencing was obtained through the semester the student was currently attending. A self-reported sociodemographic questionnaire was also applied.

### Statistical analysis

Initially, the descriptive measures of the collected variables were presented, using frequencies and percentages for categorical variables and means and standard deviations for the numerical ones. The chi-square tests were used to verify the statistical association between the measured variables and the teaching methodology. Values of *p* < 0.05 were considered significant. Data were tabulated and statistical calculations were performed using the software Statistical Package for Social Sciences (SPSS), version 23.0 (SPSS Inc., Chicago, United States)®.

### Ethical aspects

In the online application, the Free and Informed Consent form was applied through the electronic platform and made available to all participants. All necessary procedures were adopted to keep the collected data confidential. The project was submitted to the Research Ethics Committee (REC) of Unichristus.

## Results

A summary of the baseline characteristics of the study participants, which included 541 medical students, is shown in Table 1. The mean age was 23 years, and 68.5% of the participants were female. Most participants were attending the first semester, but there was a balanced distribution until the ninth semester. Most of the students (89%) had no remunerated job, and between the PBL and hybrid methodologies, 85.6% of the participants used the hybrid teaching model.

**Table 1:** Description of the sample of evaluated medical students

	n (%) or mean (SD)
How old are you?	
	23 (± 5)
What gender do you identify with?	
Male	176 (31.5%)
Female	383 (68.5%)
What semester are you attending?	
1	120 (21.6%)
2	28 (5.0%)
3	48 (8.6%)
4	50 (9.0%)
5	80 (14.4%)
6	89 (16.0%)
7	57 (10.3%)
8	69 (12.4%)
9	10 (1.8%)
10	3 (0.5%)
11	2 (0.4%)
What is your occupation?	
Only medical student	496 (89.0%)
Work beyond studying	61 (11.0%)
Learning method	
PBL	56 (14.4%)
Hybrid	332 (85.6%)

The medical students’ perceptions about online teaching and its variation between the different teaching methods are shown in Table 2. Students using the hybrid system had a higher number of daily video conferencing sessions (*p*-value < 0.001), as well as a shorter time interval between the video conferences in which they participated (*p* value = 0.02). Additionally, students using the PBL model reported longer attention span during in-person classes, although the difference was small (60 vs. 55 min, *p*-value 0.03), but there was no difference in attention span during online classes. There was no difference between the models regarding the numbers of tutoring sessions.

**Table 2:** Medical students’ impressions of online classes, in the different assessed teaching types

	PBL	Hybrid	*P* value
	N (%) or median (IQR)	N (%) or median (IQR)	
Is participating in the videoconference enjoyable for you?			
Not even a little	19 (33.9%)	70 (21.1%)	0.10
A little	14 (25.5%)	132 (39.9%)	
Moderately	11 (19.6%)	63 (19.0%)	
A lot	1 (1.8%)	16 (4.8%)	
Extremely	14 (25.5%)	132 (39.9%)	
How often/number of times a day do you participate in video conferences, on average? (numeric)	3 (2;4) Range (0–8)	4 (3;4) Range (0–12)	< 0.001
How many minutes are there, on average, between the videoconferences?	15 (10;30)	20 (15;20)	0.02
What is your average concentration span, in minutes, during a videoconference call?	30 (20;45)	30 (25;45)	0.17
What is your average concentration span, in minutes, during an in-person class?	60 (45;83)	55 (40;60)	0.03
If you have tutoring/discussion of clinical cases, how many sessions do you have per week? (numeric)	2 (2;3)	2 (2;2)	0.46
If you have tutoring, how long, in hours, does each tutoring session last on average?	3 (2;4)	3 (2;3)	0.44

**Fig. 1:**
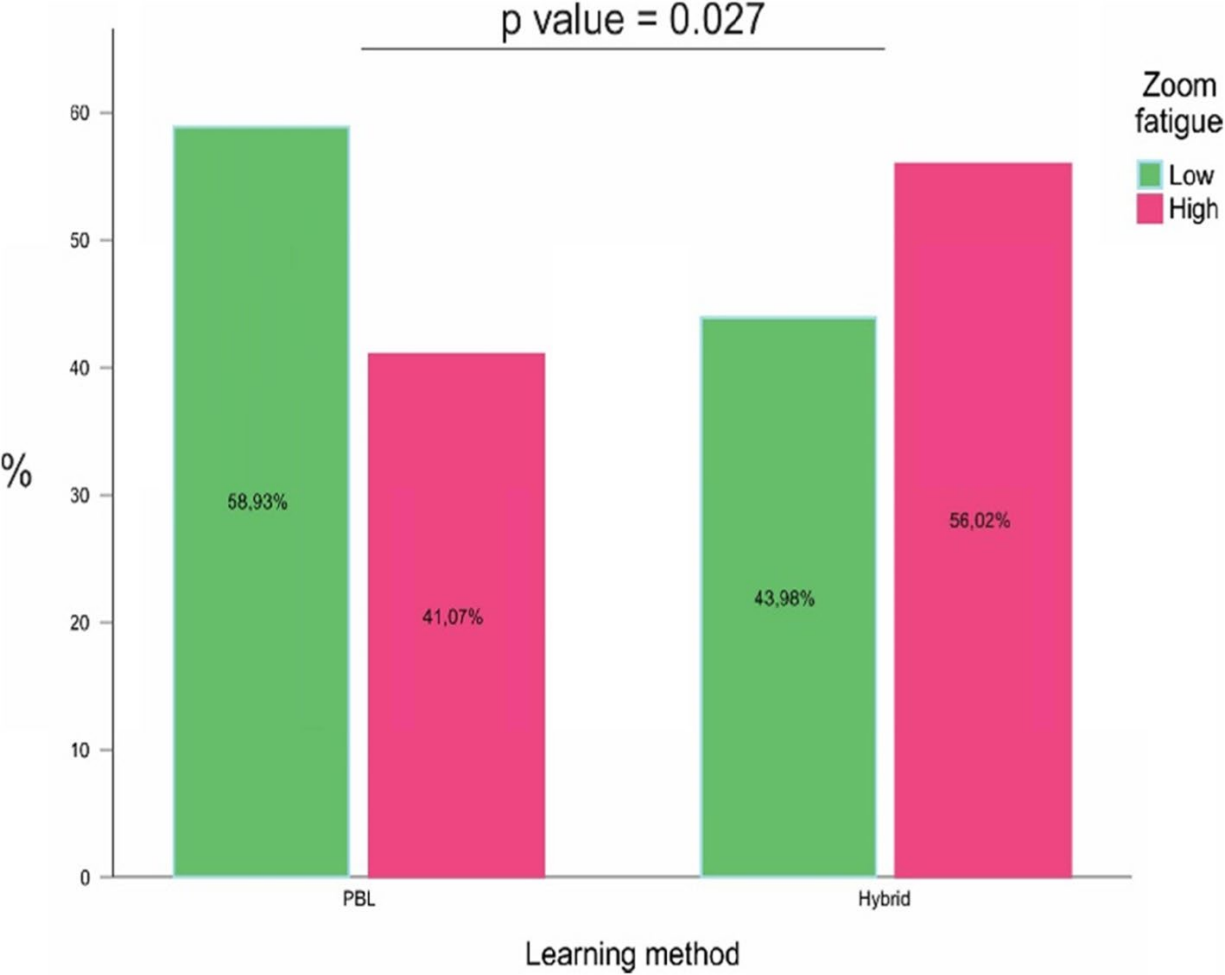
Prevalence of zoom fatigue according to the teaching method

The figure shows that the prevalence of zoom fatigue reached 56% in students using the hybrid model, *versus* 41% in those using the PBL methodology, with a statistically significant difference (Fig. 1, *p* value = 0.027). The mean prevalence of overall zoom fatigue was 48%.

When comparing the characteristics of zoom fatigue between medical students using different teaching methods, it can be observed that students using the hybrid methodology differed from PBL students by having a significantly higher frequency of feelings of wanting to be alone after a videoconference (16.9 vs. 7.1%, respectively) and needing time to be alone after a video conference (10.2 vs. 3.6%, respectively), with *p* values of 0.007 and 0.004, respectively, as shown in Table 3.

**Table 3:** Characteristics of zoom fatigue among medical students using different teaching models

	PBL	Hybrid	*P* value
	N (%)	N (%)	
After video conferences, do you feel tired?			
Not even a little	0 (0.0%)	1 (0.3%)	0.50
A little	6 (10.7%)	22 (6.6%)	
Moderately	14 (25.0%)	88 (26.5%)	
Very much	24 (42.9%)	137 (41.3%)	
Extremely	12 (21.4%)	84 (25.3%)	
After video conferences, do you feel exhausted?			
Not even a little	0 (0.0%)	9 (2.7%)	0.87
A little	7 (12.5%)	48 (14.5%)	
Moderately	14 (25.0%)	75 (22.6%)	
Very much	24 (42.9%)	122 (36.7%)	
Extremely	11 (19.6%)	78 (23.5%)	
After videoconferences, do you feel mentally drained?			
Not even a little	0 (0.0%)	4 (1.2%)	0.54
A little	4 (7.1%)	38 (11.4%)	
Moderately	7 (12.5%)	60 (18.1%)	
Very much	30 (53.6%)	124 (37.3%)	
Extremely	15 (26.8%)	106 (31.9%)	
After video conferences, is your vision blurry?			
Not even a little	11 (19.6%)	72 (21.7%)	0.66
A little	12 (21.4%)	63 (19.0%)	
Moderately	13 (23.2%)	94 (28.3%)	
Very much	13 (23.2%)	69 (20.8%)	
Extremely	7 (12.5%)	34 (10.2%)	
After video conferences, do your eyes become irritated?			
Not even a little	11 (19.6%)	53 (16.0%)	0.90
A little	15 (26.8%)	82 (24.7%)	
Moderately	7 (12.5%)	98 (29.5%)	
Very much	16 (28.6%)	61 (18.4%)	
Extremely	7 (12.5%)	38 (11.4%)	
After video conferences, do you feel pain in your eyes?			
Not even a little	17 (30.4%)	90 (27.1%)	0.85
A little	13 (23.2%)	91 (27.4%)	
Moderately	10 (17.9%)	81 (24.4%)	
Very much	11 (19.6%)	42 (12.7%)	
Extremely	5 (8.9%)	28 (8.4%)	
After video conferences, do you avoid being with other people?			
Not even a little	16 (28.6%)	79 (23.8%)	0.13
A little	17 (30.4%)	77 (23.2%)	
Moderately	13 (23.2%)	89 (26.8%)	
Very much	6 (10.7%)	60 (18.1%)	
Extremely	4 (7.1%)	27 (8.1%)	
After video conferences, do you want to be alone?			
Not even a little	16 (28.6%)	62 (18.7%)	0.007
A little	17 (30.4%)	71 (21.4%)	
Moderately	9 (16.1%)	70 (21.1%)	
Very much	10 (17.9%)	73 (22.0%)	
Extremely	4 (7.1%)	56 (16.9%)	
After video conferences, do you need time to be alone?			
Not even a little	16 (28.6%)	56 (16.9%)	0.004
A little bit	17 (30.4%)	77 (23.2%)	
Moderately	9 (16.1%)	63 (19.0%)	
Very much	10 (17.9%)	84 (25.3%)	
Extremely	4 (7.1%)	52 (15.7%)	
After video conferences, are you unmotivated to do other things?			
Not even a little	2 (3.6%)	34 (10.2%)	0.84
A little	11 (19.6%)	55 (16.6%)	
Moderately	18 (32.1%)	77 (23.2%)	
Very much	14 (25.0%)	87 (26.2%)	
Extremely	11 (19.6%)	79 23.8%)	
After video conferences, how often do you want to do nothing?			
Not even a little	0 (0.0%)	4 (1.2%)	0.78
A little	2 (3.6%)	22 (6.6%)	
Moderately	11 (19.6%)	76 (22.9%)	
Very much	34 (60.7%)	138 (42.6%)	
Extremely	9 (16.1%)	92 (27.7%)	
After video conferences, how often do you feel tired to do other things?			
Not even a little	0 (0.0%)	4 (1.2%)	0.62
A little	1 (1.8%)	17 (5.1%)	
Moderately	17 (30.4%)	96 (28.9%)	
Very much	26 (46.4%)	143 (43.1%)	
Extremely	12 (21.4%)	72 (21.7%)	
After videoconferences, do you feel emotionally drained?			
Not even a little	1 (1.8%)	30 (9.0%)	0.70
A little	13 (23.3%)	59 (17.8%)	
Moderately	12 (21.4%)	86 (25.9%)	
Very much	22 (39.3%)	87 (26.2%)	
Extremely	8 (14.3%)	70 (21.1%)	
After video conferences, do you feel irritated?			
Not even a little	5 (8.9%)	45 (13.6%)	0.87
A little	13 (23.2%)	78 (23.5%)	
Moderately	22 (39.3%)	104 (31.3%)	
Very much	12 (21.2%)	64 (19.3%)	
Extremely	4 (7.1%)	41 (12.3%)	
After video conferences, are you in a bad mood?			
Not even a little	4 (7.1%)	65 (19.6%)	0.91
A little	24 (42.9%)	87 (26.2%)	
Moderately	17 (30.4%)	101 (30.4%)	
Very much	7 (12.5%)	45 (13.6%)	
Extremely	4 (7.1%)	34 (10.2%)	

## Discussion

In this study carried out with medical students during the period of social distancing due to the COVID-19 pandemic, it was observed that the teaching modality is associated with the prevalence of zoom fatigue, with a higher prevalence identified in students using a hybrid education system, and the characteristics of social withdrawal due to zoom fatigue being the ones most often associated with this teaching modality.

The COVID-19 pandemic had the power to disrupt teaching and learning practices, thus revealing a growing ability to think and act on students and teachers in medical schools.. The challenges presented to the institutions by the COVID-19 pandemic accelerated the transformation of teaching in all sectors of education [11]. Documenting and analyzing the current effects of this change is important to learn and apply the new pedagogical principles, leading to an educational transformation that could forever change the way we teach and learn [12].

The present study showed that the average prevalence of zoom fatigue in general was 48%. The result of nearly 50% of the average prevalence of zoom fatigue is in line with reports of similar experiences in the literature that generically earned its own term, ‘zoom fatigue’, although this exhaustion also applies if you are using Google Hangouts, Skype, FaceTime or any other video calling interface [13]. The unprecedented increase in its use in response to the pandemic has launched an unofficial social experiment, showing on a population scale what has always been true: virtual interactions can be extremely difficult for the brain. A study carried out by Fauville et al. created a tool to measure this fatigue, which they called the Zoom Exhaustion and Fatigue scale, or ZEF. They conducted a survey of more than 10,000 responses that measured people’s fatigue using this ZEF scale and evaluated statistics on how much time each person spends on Zoom app in addition to demographic information [14].

The data showed the main factors involved in this fatigue were: the prolonged time on video calls, with little or no interval between them, the scarcity of non-verbal communications, increasing stress between people because they cannot naturally transmit or interpret gestures and body language considering they are only able to see the shoulders and heads of colleagues, the need to remain still in order to be visible, the “mirror anxiety” arising from the constant reflection in real time of their own image on the screen (self-awareness) and finally the “staring”, the impression that everyone else on the call is always looking at us, because the videoconference screen only shows people looking at their cameras, irrespective of who or what they are really focusing on [14].

The results also showed that the prevalence of zoom fatigue affected 56% among the students using the hybrid model, *versus* 41% using the PBL methodology, with a statistically significant difference (*p*-value = 0.027). PBL was first introduced at McMaster University in the late 1960s and, later, it was widely accepted by medical schools around the world. Simultaneously, several schools suggested modifications to the original PBL format and advocated alternative approaches that led to the birth of “hybrid” PBL (hPBL) [15]. Harvard New Pathway Curriculum has changed the scope, frequency, and format of its dialogued lectures and practical lab classes and hybridized them with active problem-based discussions (PBL) [16]. This methodology was encouraged at the beginning of the pandemic as a positive strategy for the continuity of medical education [17]. This is the model utilized in our institution since, like Malik, we believe this model has its advantages: reducing the knowledge gaps, establishing a solid education foundation of basic disciplines, encompassing different learning styles, among others. However, as students in this model are required, in addition to PBL activities, to simultaneously participate in dialogued lectures and practical activities for approximately 10 h a week, there is a higher cognitive overload when compared to the “pure” PBL model, a fact that leads these students to acquire an important degree of resilience when compared to other teaching models [18].

During the pandemic, the hPBL was carried out using educational technologies without carrying out a complete pedagogical plan, with the same workload as in-person teaching, by simply adapting the content, previously linked to the classroom, to the online environment. Pedagogical activities were usually performed live with teachers and students online on the same day and time as the in-person classes, often using videoconference platforms or applications instead of learning environments [3].

Therefore, remotely conducting lectures together with PBL (hPBL) resulted in much longer videoconferencing time when compared to teaching pure PBL, as shown in our results, leaving students with a not at all pleasant perception about the online teaching method. These facts explain the higher prevalence of zoom fatigue in this teaching model, as well as a significantly higher frequency of feelings of wanting to be alone after a videoconference (16.9 vs. 7.1%, respectively) and of needing time to be alone after a videoconference (10.2 vs. 3.6%, respectively), with *p*-values of 0.007 and 0.004, respectively, as seen in Table 3.

However, excessive participation in a videoconference when using the hybrid model is not the only explanation for the prevalence of zoom fatigue. Kaplan, 1995, in his Attention Restoration Theory (ART), postulates that individuals can reduce fatigue levels in many ways, such as a feeling of “being far away”, silencing oneself, turning off the webcam or not looking at the mirrored video of the screen. The ART also highlights that greater belonging to the environment (“compatibility”) or being involved in a task (“soft fascination”) can minimize fatigue [19].

Furthermore, for Rogelberg et al., the meetings differ from each other in several ways, which allows a more dynamic assessment of the phenomenon and expands the literature on the meetings, capturing the levels of differences between them [8, 20, 21]. Therefore, the lower prevalence of zoom fatigue in the “pure” PBL model verified in this study can be explained by the likely “compatibility” and “soft fascination” of students, inherent to the method itself, during small group tutorial sessions and the lack of lectures. It is also noteworthy that in the hPBL in the ERE, in most lectures, the teacher explains, reads slides, and the student has the right to listen with little “compatibility” and “soft fascination”. Several factors are implicated in this fact, among them, the turning off of the cameras and the fact that the interaction between the participants is a “monologue”, since, usually, when one person speaks, the others are asked to turn off the microphones, thus avoiding microphony. This contributes to the fact that video conversations are less participatory [22].

This study has some limitations. First, as this is a cross-sectional study, associations that are not causal or show reverse causality can be observed. However, it is believed that the considered exposure is prior to the outcome. Second, we used a scale that screens zoom fatigue but is not diagnostic of clinical disorders. Despite this, the validity of this scale has been demonstrated, and the risk it assesses is relevant for action taking. Third, students from more advanced semesters had a lower participation rate. Nonetheless, this variable was not statistically associated with the outcome (*p* = 0.613). Finally, the application of online questionnaires may have led to non-random selection, but almost 100% of potential students participated in the research, showing a high adherence rate.

Thus, considering that zoom fatigue may stay with us for years beyond the COVID-19 pandemic, it is important to know and provide instructions on how to reduce video conferencing fatigue. The present study suggests that the active participation of students and the number of activities are important factors to be considered. The number of students evaluated in the pure PBL modality can be considered a limiting factor in the present study and future investigations should assess the prevalence of zoom fatigue in the various active methodologies, as well as in dialogued lectures with the active involvement of participating students.

## Data Availability

The datasets used and/or analyzed during the current study are available from the corresponding author on reasonable request.

## Abbreviation

PBL: Problem-Based Learning
